# The lncRNA DLX6-AS1 promoted cell proliferation, invasion, migration and epithelial-to-mesenchymal transition in bladder cancer via modulating Wnt/β-catenin signaling pathway

**DOI:** 10.1186/s12935-019-1010-z

**Published:** 2019-11-26

**Authors:** Jinan Guo, Zhixin Chen, Hongtao Jiang, Zhou Yu, Junming Peng, Jing Xie, ZaiShang Li, Weiqing Wu, Zhiqiang Cheng, Kefeng Xiao

**Affiliations:** 1 Department of Urological Surgery, The Second Clinical Medical College of Jinan University, the First Affiliated Hospital of Southern University, Shenzhen People’s Hospital, Shenzhen, 518020 China; 2 Department of Urology, Guangzhou First People’s Hospital, The Second Affiliated Hospital of South China University of Technology, Guangzhou, 510180 China; 3 Department of Physical Examination, The Second Clinical College of Jinan University, Shenzhen People’s Hospital, Shenzhen, 518020 China; 4 Department of Pathology, the Second Clinical Medical College of Jinan University, The First Affiliated Hospital of Southern University, Shenzhen People’s Hospital, Shenzhen, 518020 China

**Keywords:** Bladder cancer, DLX6-AS1, Cell proliferation, Invasion, Migration, Epithelial-to-mesenchymal transition, Wnt/β-catenin

## Abstract

**Background:**

Bladder cancer is the most common human urological malignancies with poor prognosis, and the pathophysiology of bladder cancer involves multi-linkages of regulatory networks in the bladder cancer cells. Recently, the long noncoding RNAs (lncRNAs) have been extensively studied for their role on bladder cancer progression. In this study, we evaluated the expression of DLX6 Antisense RNA 1 (DLX6-AS1) in the cancerous bladder tissues and studied the possible mechanisms of DLX6-AS1 in regulating bladder cancer progression.

**Methods:**

Gene expression was determined by qRT-PCR; protein expression levels were evaluated by western blot assay; in vitro functional assays were used to determine cell proliferation, invasion and migration; nude mice were used to establish the tumor xenograft model.

**Results:**

Our results showed the up-regulation of DLX6-AS1 in cancerous bladder cancer tissues and bladder cell lines, and high expression of DLX6-AS1 was correlated with advance TNM stage, lymphatic node metastasis and distant metastasis. The in vitro experimental data showed that DLX6-AS1 overexpression promoted bladder cancer cell growth, proliferation, invasion, migration and epithelial-to-mesenchymal transition (EMT); while DLX6-AS1 inhibition exerted tumor suppressive actions on bladder cancer cells. Further results showed that DLX6-AS1 overexpression increased the activity of Wnt/β-catenin signaling, and the oncogenic role of DLX6-AS1 in bladder cancer cells was abolished by the presence of XAV939. On the other hand, DLX6-AS1 knockdown suppressed the activity of Wnt/β-catenin signaling, and the tumor-suppressive effects of DLX6-AS1 knockdown partially attenuated by lithium chloride and SB-216763 pretreatment. The in vivo tumor growth study showed that DLX6-AS1 knockdown suppressed tumor growth of T24 cells and suppressed EMT and Wnt/β-catenin signaling in the tumor tissues.

**Conclusion:**

Collectively, the present study for the first time identified the up-regulation of DLX6-AS1 in clinical bladder cancer tissues and in bladder cancer cell lines. The results from in vitro and in vivo assays implied that DLX6-AS1 exerted enhanced effects on bladder cancer cell proliferation, invasion and migration partly via modulating EMT and the activity of Wnt/β-catenin signaling pathway.

## Background

Bladder cancer is ninth most commonly occurred cancer in the world, and the diagnosed cases of this type of cancer was estimated to around 0.5 million annually worldwide [1, 2]. The main treatments for bladder cancer are chemotherapy, radiotherapy and surgical intervention [3–5]. Unfortunately, more than 50% patients were relapsed after medical treatments within the next 5 years, and some of patients were diagnosed at late stage, which renders difficulties for the improvement of clinical outcomes in patients with bladder cancer [6]. As far as we know, the exact molecular mechanisms underlying bladder cancer progression remain unknown, due to the complexity of the molecular regulatory networks [7]. Therefore, it is necessary to find novel markers to early diagnosis and develop novel and effective therapeutic targets to improve the clinical outcomes of bladder cancer treatment.

Long non-coding RNAs (lncRNAs) are a type of RNA with long non-coding domains and have more than 200 nucleotides [8]. Recently, multiple lines of evidence have elucidated the multi-functional role of lncRNAs in various biology processes, in particular in the pathophysiology of cancer [9]. LncRNAs can act as either oncogene or tumor suppressor to regulate cancer cell proliferation and metastasis. In the bladder cancer studies, various dysregulated lncRNAs have been identified in both bladder cancer tissues and cells. For examples, Avgeris et al. [10] screened a cohort of 176 bladder cancer patients, and identified lncRNA urothelial cancer associated 1 (UCA1) as a superior prognostic factor of disease early-relapse and progression in the bladder cancer patients. Liu et al. [11] revealed that lncRNA neuroblastoma-associated transcript 1 exerted the tumor-suppressive effects on the malignant bladder cancer cells via regulating miR-21/suppressor of cytokine signaling 6 axis. Zheng [12] found that exosome-transmitted lncRNA phosphatase and tensin homolog pseudogene 1 suppressed the progression of bladder cancer. In another study, Chen et al. [13] showed that lncRNA lymph node metastasis associated transcript 1 enhanced lymphatic metastasis via CCL2-dependent macrophage recruitment in bladder cancer. Recently, the lncRNA DLX6 Antisense RNA 1 (DLX6-AS1) has been found to be dysregulated in several types of malignant tumors, however, to our best knowledge, the expression profiles of DLX6-AS1 have been not determined in the bladder cancer yet.

The Wnt/β-catenin signaling pathway plays an important role in diverse biological processes including cell proliferation, invasion and migration [14]. Studies have shown that Wnt/β-catenin signaling participates the bladder cancer progression, and activation of Wnt/β-catenin signaling promotes bladder cancer growth and metastasis [15]. The interaction between lncRNAs and Wnt/β-catenin in bladder cancer has been addressed by several studies. Chen et al. [16] showed that lncRNA small nucleolar RNA host gene 7 knockdown exerted tumor suppressive effects on bladder cancer via targeting Wnt/β-catenin signaling; Xie et al. [17] found that lncRNA miR143HG inhibited the development of bladder cancer via inhibiting Wnt/β-catenin signaling; lncRNA cancer susceptibility candidate 2 (CACS2) knockdown enhanced bladder cancer proliferation and metastasis by potentiating the activity of Wnt/β-catenin signaling [18]. So far, whether DLX6-AS1 can interact with Wnt/β-catenin signaling pathway remains to be elucidated.

In this study, we for the first time evaluated the expression of DLX6-AS1 in the cancerous bladder tissues and bladder cancer cell lines. Further in vitro experiments were employed to elucidate the molecular mechanisms of DLX6-AS1 in regulating the cellular functions of bladder cancer cells. The present study may provide some new insights for the understanding of lncRNAs in the pathophysiology of bladder cancer.

## Materials and methods

### Collection of clinical tissues from bladder cancer patients

The experimental protocols for this study were under the approval of Ethics Committee of Shenzhen People’s Hospital, and the experiments were undertaken with the understating and written consent of all the bladder cancer patients. The cancerous bladder tissues and adjacent normal bladder tissues were collected from 54 bladder cancer patients who received surgical resection at Shenzhen People’s Hospital from 2015 to 2018. None of the patients had chemotherapy or radiotherapy before surgical resection. The histology of these specimens was examined by two independent experienced histologists. All the collected bladder cancer tissues were snap-frozen in liquid nitrogen and store in − 80 °C for further analysis. The clinical pathological data for all the patients were shown in Table 1.

**Table 1:** Association between DLX6-AS1 expression and clinicopathological features in 54 patients with bladder cancer

Characteristics	DLX6-AS1		*P* value
	Low expression (n)	High expression (n)	
Age			
≥ 60	12	17	0.1724
< 60	15	10	
Gender			
Male	15	19	0.2597
Female	12	8	
Tumor size			
< 5 cm	14	9	0.1688
≥ 5 cm	13	18	
TNM stage			
0–I	17	8	***0.014***
II/III/IV	10	19	
Tumor grade			
Low	15	9	0.1003
High	12	18	
Lymph node metastasis			
N0	18	10	***0.0293***
N1 + N2	9	17	
Distant metastasis			
No	19	11	***0.0285***
Yes	8	16	

Bolditalic values represent the statistical significance

### Cell culture

The human uroepithelial cells (SV-HUC-1) and human bladder cancer cells including 5637, J82 and T24 were all obtain from ATCC (Manassas, USA). The cells were cultured with DMEM (Sigma, St. Louis, USA) with the supplement of 10% fetal bovine serum (FBS; Sigma) in a humidified incubator under the condition of 37 °C and 5% CO_2_.

### Plasmid vectors, small interfering RNAs (siRNAs), chemical reagents treatment and cell transfection

The DLX-6AS1 overexpressing vector (pcDNA3.1-DLX6-AS1) and the control vector (pcDNA3.1) were purchased from Genescript company (Nanjing, China). The siRNAs for DLX6-AS1 (DLX6-AS1 siRNA#1 and #2) and the control scrambled siRNA as a negative control were purchased from Ribobio company (Guangzhou, China). The chemical reagents including XAV939 (Wnt/β-catenin pathway inhibitor), lithium chloride (LiCl; Wnt/β-catenin activator) and SB-216763 (Wnt/β-catenin activator) were both purchased from Sigma, and the bladder cancer cells were pre-treated with XAV939 (10 μM), LiCl (20 mM) or SB216763 (30 µM) for 24 h before further transfection studies. For the cell transfection studies, cells were transfected with the corresponding plasmids and siRNAs using the Lipofectamine 2000 reagent (Invitrogen, Carlsbad, USA) by following the manufacturer’s instructions. At 24 h after transfections, cells were prepared for further in vitro assays.

### Quantitative real-time PCR (qRT-PCR) assay to measure gene expression levels

The RNA was extracted from tumor tissues or cells using MiniBEST Universal RNA Extraction kit (Takara, Dalian, China) by following the manufacturer’s instructions. The Prime Script RT-PCR kit (Takara) was used for mRNA reverse transcription into cDNA. The corresponding gene expression was determined using SYBR green qRT-PCR kit in an ABI7900 system (Applied Biosystems, Waltham, USA). The relative expression of detected genes was calculated using the comparative Ct method with GAPDH as an internal control.

### Colony formation assay to assess bladder cancer cell growth

The treated bladder cancer cells were plated onto a 6-well plate, and the cells were grown in the full medium for 10 days with medium refreshing every 3 days. At the end of the experiments, cells were fixed with 50% ethanol and then stained with 0.5% crystal violet for 10 min at room temperature. The colony number was counted and images of the stained colonies were captured.

### Cell counting kit-8 (CCK-8) assay to assess cell proliferative capacity

The proliferative capacity of the bladder cancer cells was evaluated at 0 h, 24 h, 48 h and 72 h after transfection by using CCK-8 kit (Dojindo, Kumamoto, Japan) by following the manufacturer’s protocol.

### Transwell invasion and migration assays for the assessment of bladder cancer cell invasive and migratory abilities

The bladder cancer cell invasive and migratory capacities were evaluated using Transwell invasion and migration assays. Briefly, the treated cells were suspended in FBS-free DMEM and seed onto the upper chamber with Matrigel-coated transwell inserts (for cell invasion assay; 8 µm pore size, Millipore) or without Matrigel-coated transwell inserts (For cell migration assay, 8 µm pore size). The lower chamber was filled with full medium (served as the chemo-attractant). With a further culturing for 24 h, the invaded or migrated cells were fixed with 50% methanol followed by staining with 0.5% crystal violet at room temperature for 10 min. The number of invaded and migrated cells were assessed under a light microscope by randomly selecting five fields.

### Western blot assay to determine protein expression

Proteins from cells and tumor tissues were extracted using RIPA buffer (Sigma) with protease inhibitors, and the extracted proteins were resolved on a 10% SDS-polyacrylamide gel followed by transferring to the polyvinylidene fluoride membranes (Sigma). The membranes were then blocked with 5% non-fat milk at room temperature for 1 h before further incubating with corresponding primary antibodies against vimentin, E-cadherin, N-cadherin, β-catenin, GSK-3β, c-myc, cyclin D1 and β-actin for 12 h at 4 °C. After incubating with primary antibodies, the membranes were further incubated with horseradish peroxidase-conjugated secondary antibody for 2 h at room temperature. All these antibodies were obtained from Abcam company (Cambridge, UK). The blotting bands of corresponding proteins were visualized by using ECL kit (Thermo Fisher Scientific).

### In vivo tumor growth assay

All the animal experimental protocols were approval by the Animal Ethics Committee of Shenzhen People’s Hospital. For the construction of T24 cells with stable DLX6-AS1 knockdown, lentivirus carrying DLX6-AS1 shRNA or control shRNA (GeneChem, Shanghai, China) were infected into T24 cells with the selection by puromycin. For the animal studies, the 5-week old male nude mice (each group had 5 mice) were inoculated with the corresponding T24 cells (cells in prepared in 50 µl PBS mixed with Matrigel in a ratio of 4:1 v/v) with control shRNA expression or DLX6-AS1 shRNA expression, and after cell injections, the tumor volume was evaluated every week for 5 weeks. The tumor volume was calculated using formula: length × length × width/2. At the end of the experiments, the animals were killed by cervical dislocation, and the tumors were collected for weight assessment and in vitro assays.

### Statistical analysis

The data collected in this study was analyzed using GraphPad Prism 6.0 (GraphPad Software, La Jolla, USA). All the data were expressed as mean ± standard deviation. The categorical data was analyzed by the Chi-square test. Significant differences for the continuous data were analyzed using Student’s *t* test or one-way ANOVA. P < 0.05 was considered to be statistically significant.

## Results

### Up-regulation of DLX6-AS1 in bladder cancer tissues and cell lines

**Fig. 1:**
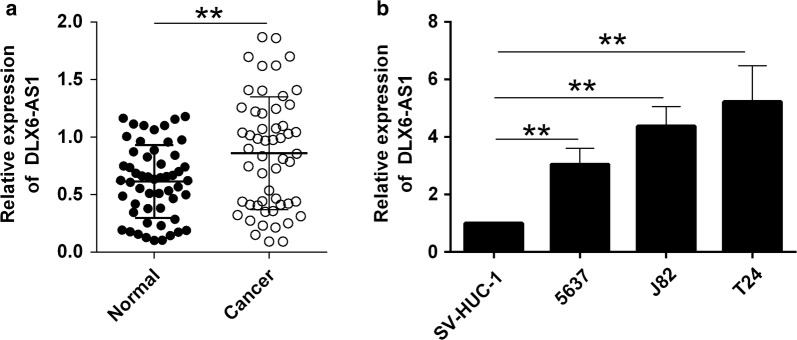
Up-regulation of DLX6-AS1 in bladder cancer tissues and cell lines. **a** Analysis of DLX6-AS1 expression by qRT-PCR in adjacent normal bladder tissues and bladder cancer tissues from 54 patients. **b** Analysis of DLX6-AS1 expression by qRT-PCR in human uroepithelial cells and bladder cancer cell lines (n = 3). Significant differences between different groups were shown as **P < 0.01

The expression of DLX6-AS1 was first determined in the clinical sample tissues from 54 patients with bladder cancer. As illustrated in Fig. 1a, the DLX6-AS1 was significantly up-regulated in the cancerous bladder tissues when compared to the adjacent normal bladder tissues (Fig. 1a). Based on the median values of DLX6-AS1 expression in cancerous bladder tissues, the expression of DLX6-AS1 was divided into “low expression” and “high expression” groups, and Chi-square test analysis revealed that high expression of DLX6-AS1 was positively correlated with advanced TNM stage, lymph node metastasis and distant metastasis (Table 1), and DLX6-AS1 expression had not significant correlation with other parameters including gender, tumor size and tumor grade (Table 1). The analysis of DLX6-AS1 expression in the normal uroepithelial cells and bladder cancer cell lines revealed that DLX6-AS1 was markedly up-regulated in the bladder cancer cells lines when compared to normal uroepithelial cells (Fig. 1b).

### Overexpression of DLX6-AS1 promoted bladder cancer cell proliferation, invasion, migration and EMT

**Fig. 2:**
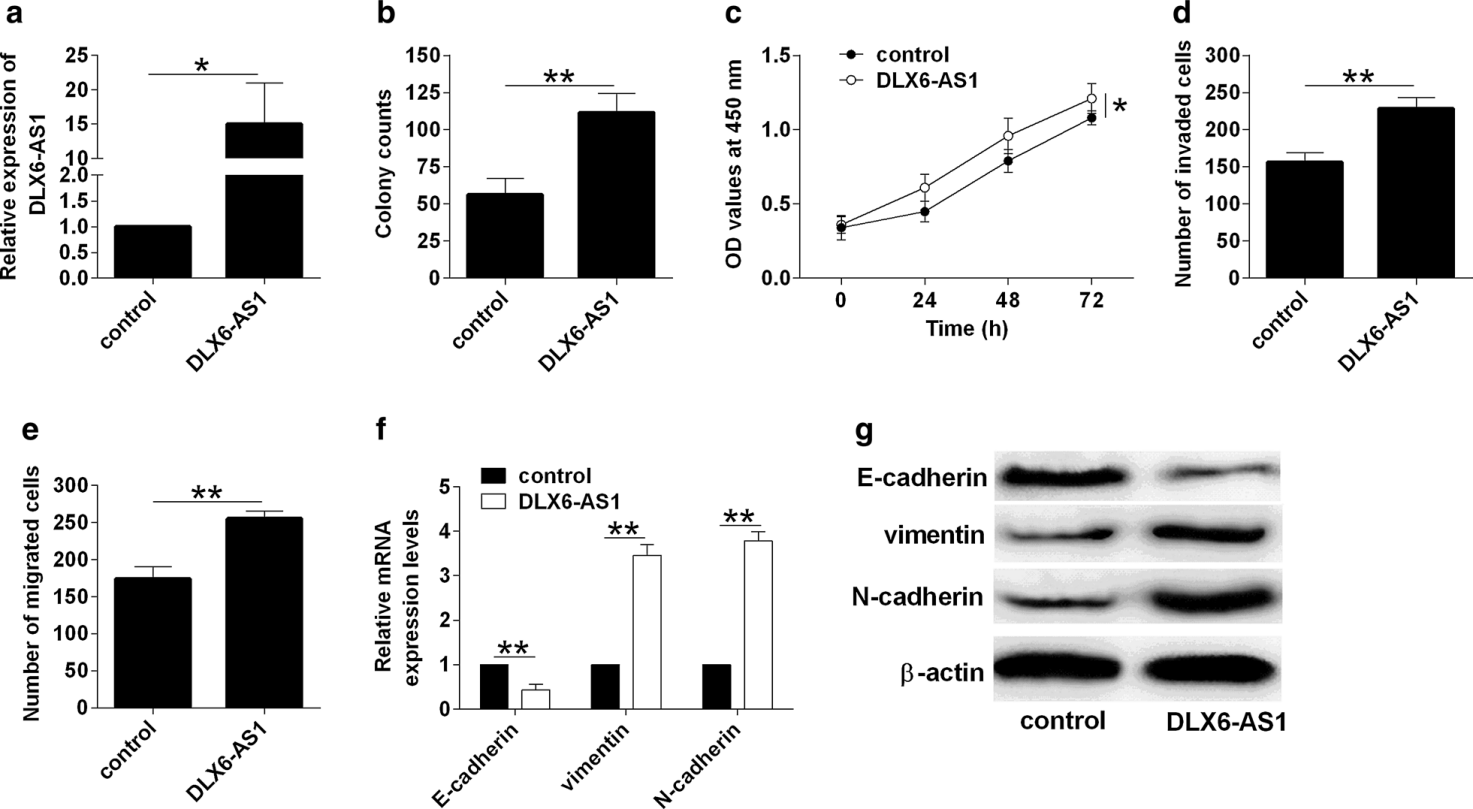
Overexpression of DLX6-AS1 promoted bladder cancer cell proliferation, invasion, migration and EMT. **a** Analysis of DLX6-AS1 expression by qRT-PCR in J82 cells after being transfected with control vector or DLX6-AS1 overexpressing vector. **b** Analysis of cell growth by colony formation assay, **c** analysis of cell proliferation by CCK-8 assay, **d** analysis of cell invasion by transwell invasion assay and **e** analysis of cell migration by transwell migration assay in J82 cells after being transfected with control vector or DLX6-AS1 overexpression vector. **f**, **g** Analysis of E-cadherin, vimentin and N-cadherin expression by qRT-PCR and western blot assays in J82 cells after being transfected with control vector or DLX6-AS1 overexpression vector. N = 3; significant differences between different groups were shown as *P < 0.05 and **P < 0.01

The effects of DLX6-AS1 on the cellular function of bladder cancer cells were determined by in vitro assays. The transient overexpression of DLX6-AS1 in J82 cells were achieved by DLX6-AS1 overexpressing vector transfection, and the transfection of DLX6-AS1 overexpressing vector significantly enhanced DLX6-AS1 expression in J82 cells when compared to control vector transfection (Fig. 2a). The cell proliferation were evaluated in J82 cells with/without DLX6-AS1 overexpression, and overexpression of DLX6-AS1 significantly increased the number of colonies and the proliferative index of J82 cells when compared to control group (Fig. 2b, c). Further transwell invasion and migration assays showed that up-regulation of DLX6-AS1 caused an increase in the number of invaded and migrated J82 cells when compared to normal group (Fig. 2d, e). The analysis of EMT-related markers showed that DLX6-AS1 overexpression increased the mRNA and protein levels of vimentin and N-cadherin, but decreased E-cadherin mRNA and protein levels in J82 cells when compared to control group (Fig. 2f, g).

### Knockdown of DLX6-AS1 inhibited bladder cancer cell proliferation, invasion, migration and EMT

**Fig. 3:**
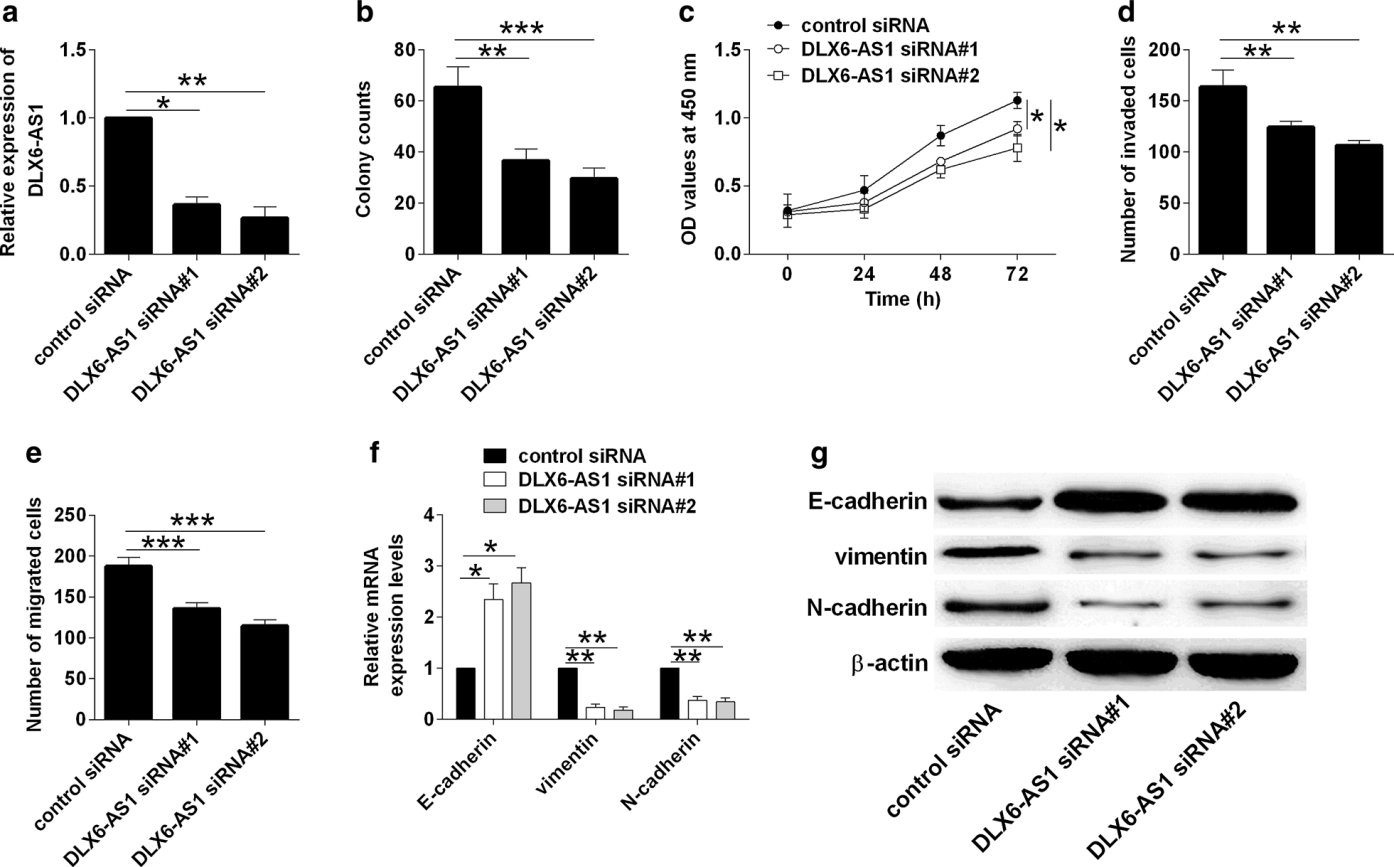
Knockdown of DLX6-AS1 inhibited bladder cancer cell proliferation, invasion, migration and EMT. **a** Analysis of DLX6-AS1 expression by qRT-PCR in J82 cells after being transfected with control siRNA or DLX6-AS1 siRNA. **b** Analysis of cell growth by colony formation assay, **c** analysis of cell proliferation by CCK-8 assay, **d** analysis of cell invasion by transwell invasion assay and **e** analysis of cell migration by transwell migration assay in T24 cells after being transfected with control siRNA or DLX6-AS1 siRNA. **f**, **g** Analysis of E-cadherin, vimentin and N-cadherin expression by qRT-PCR and western blot assays in T24 cells after being transfected with control siRNA o DLX6-AS1 siRNA. N = 3; significant differences between different groups were shown as *P < 0.05 and **P < 0.01

The knockdown effects of DLX6-AS1 on bladder cancer cellular function were determined by loss-of-function study. As shown in Fig. 3a, transfection with DLX6-AS1 siRNAs dramatically suppressed DLX6-AS1 expression in T24 cells when compared to control siRNA group (Fig. 3a). Consistently, knockdown of DLX6-AS1 reduced the number of colonies, inhibited cell proliferation, invasion and migration in T24 cells when compared to control siRNA group (Fig. 3b–e). Further western blot assay showed that knockdown of DLX6-AS1 caused a down-regulation of vimentin and N-cadherin, and an up-regulation of E-cadherin (Fig. 3f, g).

### Overexpression of DLX6-AS1 enhanced Wnt/β-catenin signaling in J82 cells

**Fig. 4:**
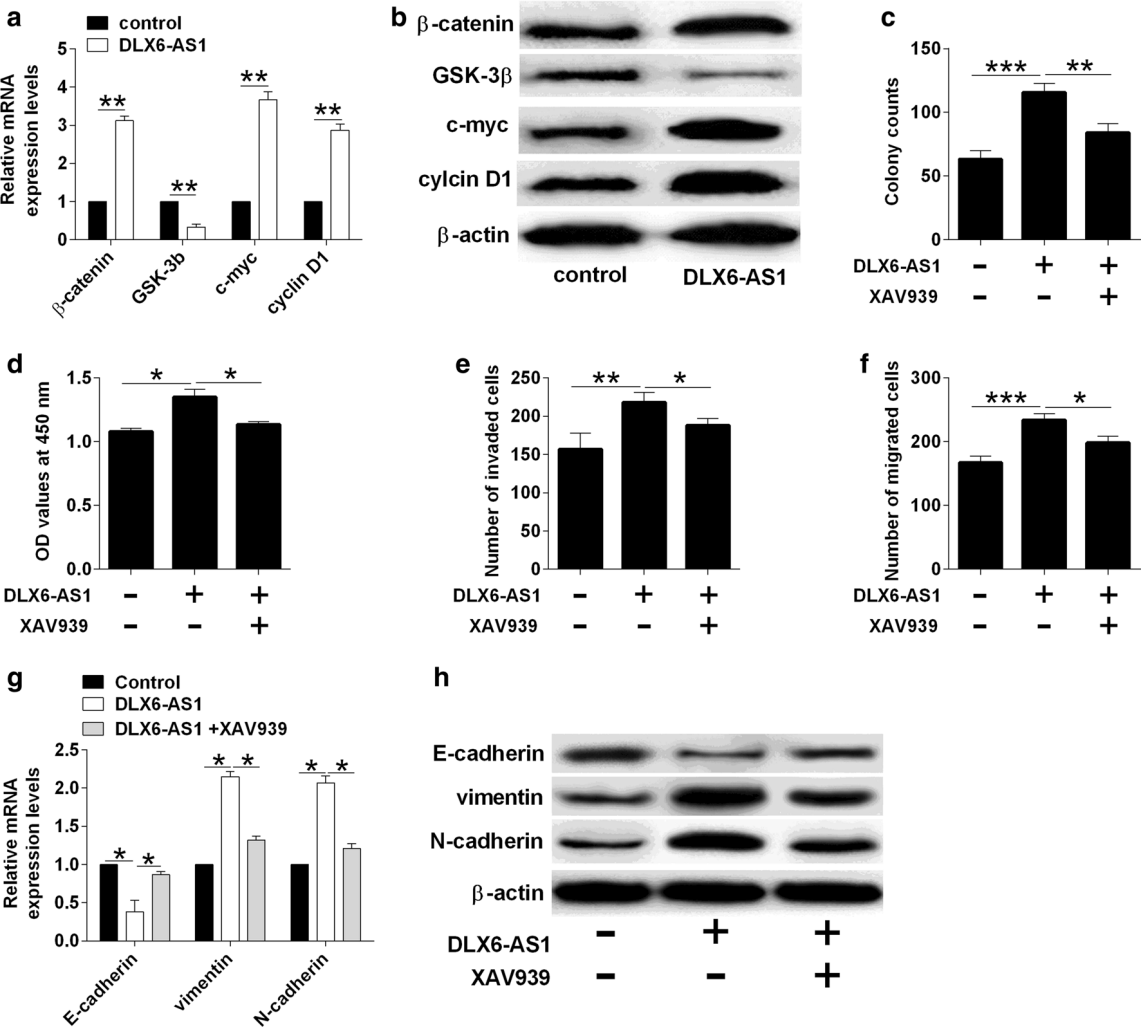
Overexpression of DLX6-AS1 enhanced Wnt/β-catenin signaling in J82 cells. **a** Analysis of β-catenin, GSK-3β, c-myc and cyclin D1 mRNA expression by qRT-PCR in J82 cells after being transfected with control vector or DLX6-AS1 overexpressing vector. **b** Analysis of β-catenin, GSK-3β, c-myc and cyclin D1 protein expression by western blot in J82 cells after being transfected with control vector or DLX6-AS1 overexpressing vector. **c** Analysis of cell growth by colony formation assay, **d** analysis of cell proliferation by CCK-8 assay, **e** analysis of cell invasion by transwell invasion assay, **f** analysis of cell migration by transwell migration assay, **g** and **h** analysis of E-cadherin, vimentin and N-cadherin expression by qRT-PCR and western blot assays in vehicle or XAV939-treated J82 cells after being transfected with control vector or DLX6-AS1 overexpressing vector. N = 3; significant differences between different groups were shown as *P < 0.05, **P < 0.01 and ***P < 0.001

Wnt/β-catenin signaling has been demonstrated to play an important role in bladder cancer progression [15] and studies indicated that DLX6-AS1 regulated bladder cancer progression via Wnt/β-catenin signaling in pancreatic cancer [19]. Thus, further qRT-PCR and western blot assays revealed that DLX6-AS1 overexpression induced up-regulation of β-catenin, c-myc and cyclin D1, but down-regulation of GSK-3β (Fig. 4a, b). To confirm the involvement of Wnt/β-catenin signaling in DLX6-AS1 mediated effects in J82 cells, the J82 cells were pre-treated with the inhibitor (XAV939) of Wnt/β-catenin signaling, and the presence of XAV939 partially repressed the enhancing effects of DLX6-AS1 overexpression on J82 cell growth, proliferation, invasion and migration (Fig. 4c–f). Moreover, XAV939 treatment also counteracted the promoting effects of DLX6-AS1 overexpression on the EMT of J82 cells (Fig. 4g, h).

### Knockdown of DLX6-AS1 inhibited Wnt/β-catenin signaling in T24 cells

**Fig. 5:**
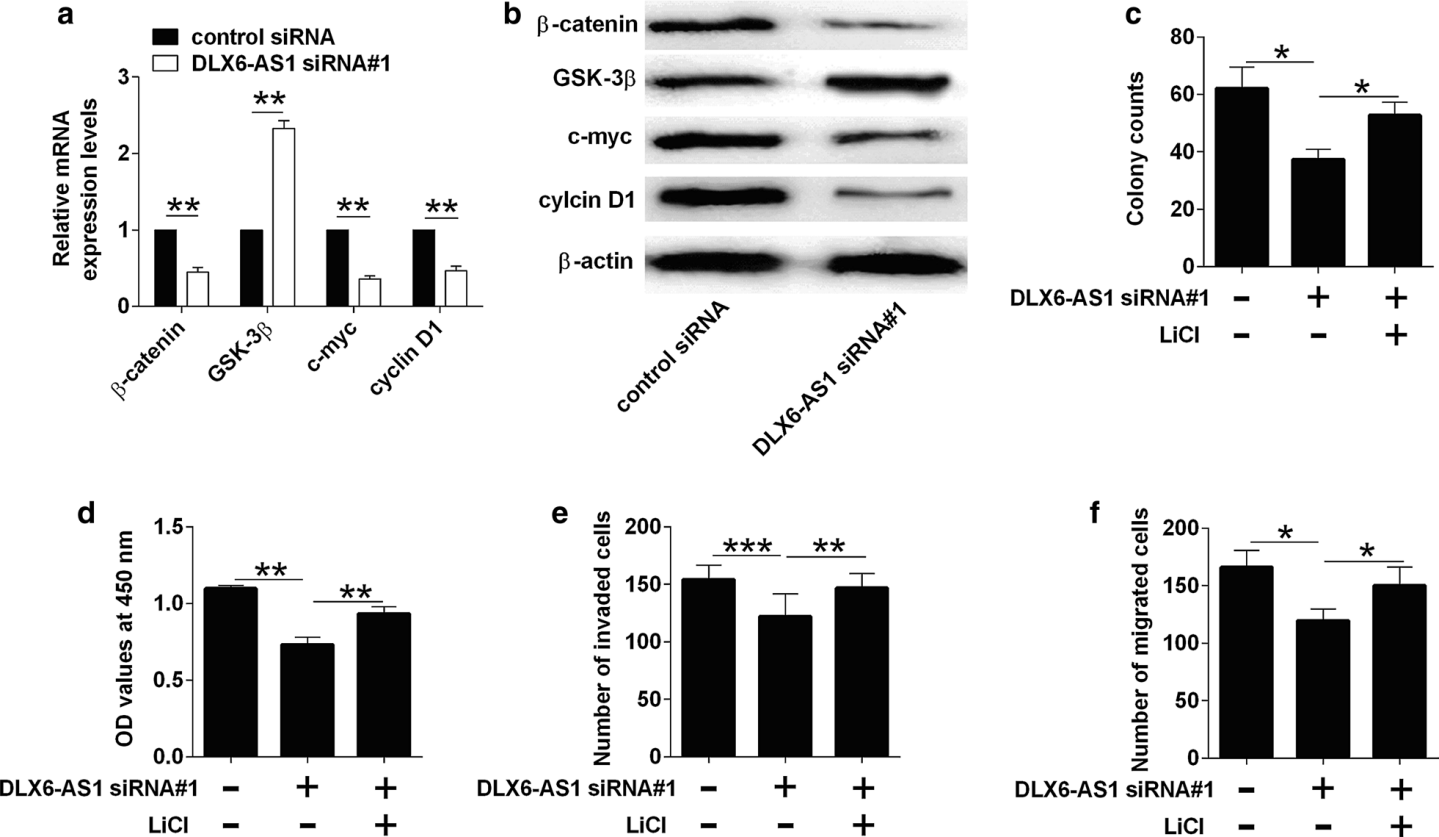
Knockdown of DLX6-AS1 inhibited Wnt/β-catenin signaling in T24 cells. **a** Analysis of β-catenin, GSK-3β, c-myc and cyclin D1 mRNA expression by qRT-PCR in T24 cells after being transfected with control siRNA or DLX6-AS1 siRNA. **b** Analysis of β-catenin, GSK-3β, c-myc and cyclin D1 protein expression by western blot in T24 cells after being transfected with control siRNA or DLX6-AS1 siRNA. **c** Analysis of cell growth by colony formation assay, **d** analysis of cell proliferation by CCK-8 assay, **e** analysis of cell invasion by transwell invasion assay and **f** analysis of cell migration by transwell migration assay in vehicle or LiCl-treated T24 cells after being transfected with control siRNA or DLX6-AS1 siRNA. N = 3; significant differences between different groups were shown as *P < 0.05, **P < 0.01 and ***P < 0.001

On the other hand, knockdown of DLX6-AS1 suppressed the mRNA and protein expression of β-catenin, c-myc and cyclin D1, but enhanced the expression of GSK-3β (Fig. 5a, b). Furthermore, the treatment with Wnt/β-catenin signaling activator, LiCl, attenuated the inhibitory effects of DLX6-AS1 on tumor cell growth, proliferation, invasion and migration in T24 cells (Fig. 5c, d). Consistently, SB-216763, a potent GSK-3β inhibitor, significantly attenuated the inhibitory effects of DLX6-AS1 knockdown on the cell proliferation, invasion and migration of T24 cells (Additional file 1: Figure S1).

### Knockdown of DLX6-AS1 inhibited in vivo tumor growth

**Fig. 6:**
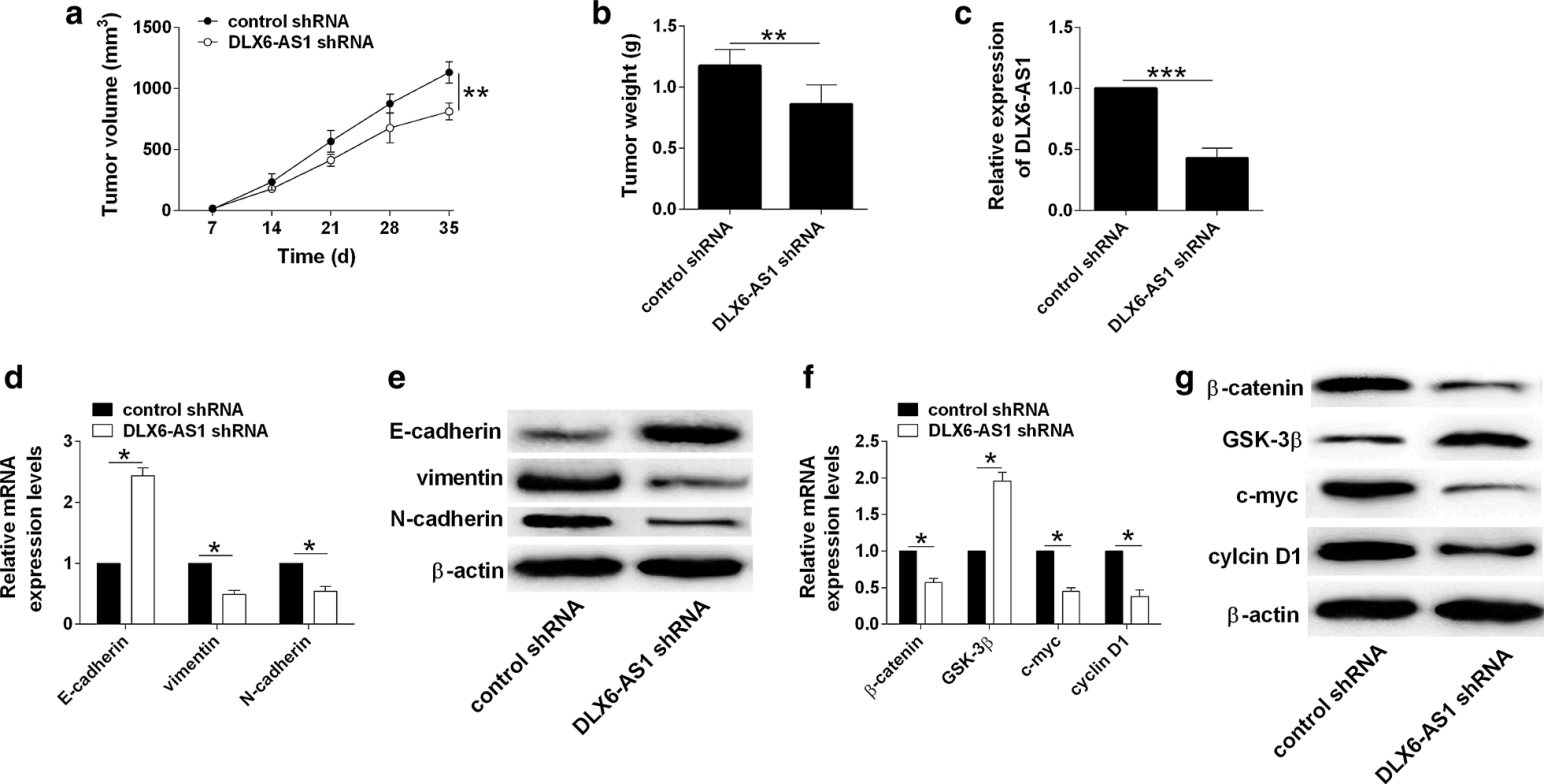
Knockdown of DLX6-AS1 inhibited in vivo tumor growth. **a** Analysis of tumor growth in nude mice inoculated with T24 cells (transfected control shRNA or DLX6-AS1 shRNA). **b** Analysis of tumor weight of the dissected tumor tissues from control shRNA and DLX6-AS1 shRNA groups. **c** Analysis of DLX6-AS1 expression by qRT-PCR in tumor tissues from control shRNA and DLX6-AS1 shRNA groups. **d**, **e** Analysis of E-cadherin vimentin, and N-cadherin mRNA and protein expression by qRT-PCR and western blot, respectively, in tumor tissues from control shRNA and DLX6-AS1 shRNA group. **f**, **g** Analysis of β-catenin, GSK-3β, c-myc and cyclin D1 mRNA and protein expression by qRT-PCR and western blot, respectively, in tumor tissues from control shRNA and DLX6-AS1 shRNA group. N = 5; significant differences between different groups were shown as *P < 0.05, **P < 0.01 and ***P < 0.001

The knockdown effects of DLX6-AS1 on the in vivo tumor growth were assessed on a xenograft nude mice model. As shown in Fig. 6a, the tumor growth was significantly repressed in the DLX6-AS1 shRNA group when compared to control shRNA group (Fig. 6a). Furthermore, the weight of dissected tumor tissues from DLX6-AS1 group was reduced when compared to that from control shRNA group (Fig. 6b). The analysis of DLX6-AS1 by qRT-PCR showed that DLX6-AS1 expression was down-regulated in DLX6-AS1 shRNA group when compared to control shRNA group (Fig. 6c). Knockdown of DLX6-AS1 down-regulated vimentin and N-cadherin, but up-regulated E-cadherin in the tumor tissues (Fig. 6d, e). More importantly, the activity of Wnt/β-catenin signaling was significantly repressed in the DLX6-AS1 shRNA group when compared to control shRNA group (Fig. 6f, g).

## Discussion

Bladder cancer is the most common human urological malignancies with poor prognosis, and the pathophysiology of bladder cancer involves multi-linkages of regulatory networks in the bladder cancer cells [6]. Therefore, discovery of novel biomarkers for diagnosis and explore new therapeutic approaches are urgent for improving the treatment for bladder cancer. In our study, we identified the up-regulation of DLX6-AS1 in cancerous bladder cancer tissues and bladder cell lines, and high expression of DLX6-AS1 was correlated with advance TNM stage, lymphatic node metastasis and distant metastasis. The gain- and loss-of-function studies revealed the oncogenic role of DLX6-AS1 in bladder cancer cells, and DLX6-AS1 also promoted EMT and enhanced Wnt/β-catenin signaling in bladder cancer cells. Collectively, the present study may imply the novel actions of DLX6-AS1 in the bladder cancer progression.

DLX6-AS1 is located on chr7:96955141-97014065 and has been well-documented in several cancer studies. In the liver cancer, DLX6-AS1 could enhance WEE1 expression via targeting miR-424-5p to aggregate the liver cancer progression [20]; in addition, DLX6-AS1 promoted liver cancer carcinogenesis via targeting the miR-203a/matrix metalloproteinase-2 axis [21]. In the lung cancer, DLX6-AS1 was found to be up-regulated and knockdown of DLX6-AS1 inhibited lung cancer progression via suppressing PRR1 expression and up-regulating miR-144 expression [22, 23]. DLX6-AS1 was also found to promote renal cell carcinoma progression via miR-26a/phosphatase and tensin homologue axis [24], and in the pancreatic cancer, An et al., showed that DLX6-AS1 functioned as an endogenous RNA for miR-181b to promote cancer cell proliferation and invasion [25]. In the glioma, DLX6-AS1 accelerated the carcinogenesis via suppressing miR-197-5p to suppress E2F1 [26]. In consistent with these previous studies, the present study showed that DLX6-AS1 overexpression enhanced bladder cancer cell proliferation, invasion and migration, while DLX6-AS1 knockdown suppressed bladder cancer cell progression. All these data may imply the oncogenic role of DLX6-AS1 in the bladder cancer.

EMT is an important process in the progression of bladder cancer, and enhanced EMT has been linked to the accelerated metastasis of bladder cancer [27]. The EMT process could be influenced by various dysregulated lncRNAs such as lncRNA TP73 Antisense RNA 1 [28], UCA1 [29], long intergenic non-protein coding RNA, regulator of reprogramming [30], taurine up-regulated 1 [31] and so on. In the present study, we consistently revealed that DLX6-AS1 exerted enhanced effects on the EMT of bladder cancer cells, which may indicate that the enhanced effects of DLX6-AS1 on bladder cancer cell invasion and migration may be related to EMT.

Previous studies have underscored the importance of Wnt/β-catenin signaling pathway in the bladder cancer progression [32]. Therefore, to elucidate the molecular mechanisms of DLX6-AS1 in regulating bladder cancer progression, we focused on the Wnt/β-catenin signaling pathway, and determined the expression of several key factors including β-catenin, GSK-3β c-myc and cyclin D1. Our data revealed that DLX6-AS1 enhanced the Wnt/β-catenin signaling activities; and presence of XAV393 or LiCl partially abolished the respective effects of DLX6-AS1 overexpression or knockdown on the bladder cancer cell proliferation, invasion and migration. Previous studies showed that DXL6-AS1 aggravated osteosarcoma stemness via Wnt/β-catenin signaling [33]; DLX6-AS1 could also interact with Wnt/β-catenin signaling to promote tumorigenesis in pancreatic cancer [19]. Collectively, our data may imply that DLX6-AS1-mediated effects on bladder cancer cell proliferation, invasion and migration may involve Wnt/β-catenin signaling pathway.

The regulatory actions of DLX6-AS1 on the bladder cancer progression may involve other targets. Previous studies have implicated that DLX6-AS1 exerted oncogenic actions in various types of cancers via interacting with different miRNAs such as miR-577 [34], miR-203a [21], miR-129-5p [33] and miR-204-5p [35]. In addition, in the liver cancer stem cells, DLX6-AS1 knockdown inhibited cell adhesion molecule 1 promoter methylation, which led to the inhibition of tumorigenesis [36]. Recently, DLX6-AS1 was found to modulate gastric cancer progression via FUS-regulated MAP4K1 [37]. Whether these interactions exist in bladder cancer still require further investigations.

In this study, we should also pay attention to the possible limitations. First of all, the sample size of the recruited patients is relatively small in our study, and further study may include more patients for analysis to confirm the current findings. For the aspect of mechanistic role of DLX6-AS1, it is possible that DLX6-AS1 may also act an endogenous RNA for miRNAs in the bladder cancer, which may require further investigation. Whether DLX6-AS1 could serve as a prognostic marker for bladder cancer may require the overall survival data of the bladder cancer patients in the future follow-up studies.

## Conclusions

In summary, the present study for the first time identified the up-regulation of DLX6-AS1 in clinical bladder cancer tissues and in bladder cancer cell lines. The results from in vitro and in vivo assays implied that DLX6-AS1 exerted enhanced effects on bladder cancer cell proliferation, invasion and migration partly via modulating EMT and the activity of Wnt/β-catenin signaling pathway. Our study proposed a novel oncogenic action of DLX6-AS1 in bladder cancer, and targeting of DLX6-AS1 may represent a potential therapeutic target for bladder cancer, which still requires more detailed investigations.

## Supplementary information

**Additional file 1: Figure S1.** SB-216763 attenuated the effects of DLX6-AS1 knockdown on the proliferation, invasion and migration of T24 cells. (A) Analysis of cell growth by colony formation assay, (B) analysis of cell proliferation by CCK-8 assay, (C) analysis of cell invasion by transwell invasion assay and (D) analysis of cell migration by transwell migration assay in vehicle or SB-216763-treated T24 cells after being transfected with control siRNA or DLX6-AS1 siRNA. N = 3; significant differences between different groups were shown as *P < 0.05 and **P < 0.01.

## Data Availability

All the data in the manuscript are available upon reasonable request.

## References

[1] Gourd E (2018). Neoadjuvant pembrolizumab in bladder cancer. Lancet Oncol.

[2] Miyamoto DT, Mouw KW, Feng FY, Shipley WU, Efstathiou JA (2018). Molecular biomarkers in bladder preservation therapy for muscle-invasive bladder cancer. Lancet Oncol.

[3] Cattrini C, Boccardo F (2018). Atezolizumab and bladder cancer: facing a complex disease. Lancet (London, England).

[4] Dreicer R (2017). New option for cisplatin-ineligible urothelial cancer. Lancet Oncol.

[5] Seiler R, Thalmann GN (2018). Robot-assisted versus open cystectomy. Lancet (London, England).

[6] Kamat AM, Hahn NM, Efstathiou JA, Lerner SP, Malmstrom PU, Choi W, Guo CC, Lotan Y, Kassouf W (2016). Bladder cancer. Lancet (London, England).

[7] Dobruch J, Daneshmand S, Fisch M, Lotan Y, Noon AP, Resnick MJ, Shariat SF, Zlotta AR, Boorjian SA (2016). Gender and bladder cancer: a collaborative review of etiology, biology, and outcomes. Eur Urol.

[8] Quan J, Pan X, Zhao L, Li Z, Dai K, Yan F, Liu S, Ma H, Lai Y (2018). LncRNA as a diagnostic and prognostic biomarker in bladder cancer: a systematic review and meta-analysis. OncoTargets Ther.

[9] Wieczorek E, Reszka E (2018). mRNA, microRNA and lncRNA as novel bladder tumor markers. Clin Chim Acta.

[10] Avgeris M, Tsilimantou A, Levis PK, Rampias T, Papadimitriou MA, Panoutsopoulou K, Stravodimos K, Scorilas A (2019). Unraveling UCA1 lncRNA prognostic utility in urothelial bladder cancer. Carcinogenesis.

[11] Liu Z, Xie D, Zhang H (2018). Long noncoding RNA neuroblastoma-associated transcript 1 gene inhibits malignant cellular phenotypes of bladder cancer through miR-21/SOCS6 axis. Cell Death Dis.

[12] Zheng R, Du M, Wang X, Xu W, Liang J, Wang W, Lv Q, Qin C, Chu H, Wang M (2018). Exosome-transmitted long non-coding RNA PTENP1 suppresses bladder cancer progression. Mol Cancer.

[13] Chen C, He W, Huang J, Wang B, Li H, Cai Q, Su F, Bi J, Liu H, Zhang B (2018). LNMAT1 promotes lymphatic metastasis of bladder cancer via CCL2 dependent macrophage recruitment. Nat Commun.

[14] Krishnamurthy N, Kurzrock R (2018). Targeting the Wnt/beta-catenin pathway in cancer: update on effectors and inhibitors. Cancer Treat Rev.

[15] Garg M, Maurya N (2019). WNT/beta-catenin signaling in urothelial carcinoma of bladder. World J Nephrol.

[16] Chen Y, Peng Y, Xu Z, Ge B, Xiang X, Zhang T, Gao L, Shi H, Wang C, Huang J (2019). Knockdown of lncRNA SNHG7 inhibited cell proliferation and migration in bladder cancer through activating Wnt/beta-catenin pathway. Pathol Res Pract.

[17] Xie H, Huang H, Huang W, Xie Z, Yang Y, Wang F (2019). LncRNA miR143HG suppresses bladder cancer development through inactivating Wnt/beta-catenin pathway by modulating miR-1275/AXIN2 axis. J Cell Physiol.

[18] Pei Z, Du X, Song Y, Fan L, Li F, Gao Y, Wu R, Chen Y, Li W, Zhou H (2017). Down-regulation of lncRNA CASC2 promotes cell proliferation and metastasis of bladder cancer by activation of the Wnt/beta-catenin signaling pathway. Oncotarget.

[19] Yang J, Ye Z, Mei D, Gu H, Zhang J (2019). Long noncoding RNA DLX6-AS1 promotes tumorigenesis by modulating miR-497-5p/FZD4/FZD6/Wnt/beta-catenin pathway in pancreatic cancer. Cancer Manag Res.

[20] Li D, Tang X, Li M, Zheng Y (2019). Long noncoding RNA DLX6-AS1 promotes liver cancer by increasing the expression of WEE1 via targeting miR-424-5p. J Cell Biochem.

[21] Zhang L, He X, Jin T, Gang L, Jin Z (2017). Long non-coding RNA DLX6-AS1 aggravates hepatocellular carcinoma carcinogenesis by modulating miR-203a/MMP-2 pathway. Biomed Pharmacother.

[22] Huang Y, Ni R, Wang J, Liu Y (2019). Knockdown of lncRNA DLX6-AS1 inhibits cell proliferation, migration and invasion while promotes apoptosis by downregulating PRR11 expression and upregulating miR-144 in non-small cell lung cancer. Biomed Pharmacother.

[23] Li J, Li P, Zhao W, Yang R, Chen S, Bai Y, Dun S, Chen X, Du Y, Wang Y (2015). Expression of long non-coding RNA DLX6-AS1 in lung adenocarcinoma. Cancer Cell Int.

[24] Zeng X, Hu Z, Ke X, Tang H, Wu B, Wei X, Liu Z (2017). Long noncoding RNA DLX6-AS1 promotes renal cell carcinoma progression via miR-26a/PTEN axis. Cell cycle (Georgetown, Tex).

[25] An Y, Chen XM, Yang Y, Mo F, Jiang Y, Sun DL, Cai HH (2018). LncRNA DLX6-AS1 promoted cancer cell proliferation and invasion by attenuating the endogenous function of miR-181b in pancreatic cancer. Cancer Cell Int.

[26] Li X, Zhang H, Wu X (2019). Long noncoding RNA DLX6-AS1 accelerates the glioma carcinogenesis by competing endogenous sponging miR-197-5p to relieve E2F1. Gene.

[27] Monteiro-Reis S, Lobo J, Henrique R, Jeronimo C (2019). Epigenetic mechanisms influencing epithelial to mesenchymal transition in bladder cancer. Int J Mol Sci..

[28] Tuo Z, Zhang J, Xue W (2018). LncRNA TP73-AS1 predicts the prognosis of bladder cancer patients and functions as a suppressor for bladder cancer by EMT pathway. Biochem Biophys Res Commun.

[29] Luo J, Chen J, Li H, Yang Y, Yun H, Yang S, Mao X (2017). LncRNA UCA1 promotes the invasion and EMT of bladder cancer cells by regulating the miR-143/HMGB1 pathway. Oncol Lett.

[30] Chen Y, Peng Y, Xu Z, Ge B, Xiang X, Zhang T, Gao L, Shi H, Wang C, Huang J (2017). LncROR promotes bladder cancer cell proliferation, migration, and epithelial–mesenchymal transition. Cell Physiol Biochem.

[31] Tan J, Qiu K, Li M, Liang Y (2015). Double-negative feedback loop between long non-coding RNA TUG1 and miR-145 promotes epithelial to mesenchymal transition and radioresistance in human bladder cancer cells. FEBS Lett.

[32] Schulz WA (2006). Understanding urothelial carcinoma through cancer pathways. Int J Cancer.

[33] Zhang RM, Tang T, Yu HM, Yao XD (2018). LncRNA DLX6-AS1/miR-129-5p/DLK1 axis aggravates stemness of osteosarcoma through Wnt signaling. Biochem Biophys Res Commun.

[34] Zhou FR, Pan ZP, Shen F, Huang LQ, Cui JH, Cai K, Guo XL (2019). Long noncoding RNA DLX6-AS1 functions as a competing endogenous RNA for miR-577 to promote malignant development of colorectal cancer. Eur Rev Med Pharmacol Sci.

[35] Liang Y, Zhang CD, Zhang C, Dai DQ (2019). DLX6-AS1/miR-204-5p/OCT1 positive feedback loop promotes tumor progression and epithelial–mesenchymal transition in gastric cancer. Gastric Cancer.

[36] Wu DM, Zheng ZH, Zhang YB, Fan SH, Zhang ZF, Wang YJ, Zheng YL, Lu J (2019). Down-regulated lncRNA DLX6-AS1 inhibits tumorigenesis through STAT3 signaling pathway by suppressing CADM1 promoter methylation in liver cancer stem cells. J Exp Clin Cancer Res.

[37] Wu Q, Ma J, Meng W, Hui P (2019). DLX6-AS1 promotes cell proliferation, migration and EMT of gastric cancer through FUS-regulated MAP4K1. Cancer Biol Ther.

